# The Limits and Contributions of Formal Support: Service Providers’ Perspectives on Balancing Formal and Natural Support for People with Disabilities and their Families in Canada

**DOI:** 10.1007/s10882-023-09944-2

**Published:** 2024-01-02

**Authors:** Reshma Parvin Nuri, Caitlin Piccone, Navjit Gaurav, Donna Thomson, Rebecca Pauls, Linda Perry, Heather Michelle Aldersey

**Affiliations:** 1 School of Rehabilitation Therapy, Queen’s University, Louise D. Acton Building, 31 George Street, Kingston, ON K7L 3N6 Canada; 2 Family Partner, Ottawa, ON Canada; 3 Planned Lifetime Advocacy Network, 101-1001 West Broadway, Vancouver, BC V6H 4E4 Canada; 4 Vela Microboard Association of British Columbia, Langley, BC V3A 5M7 Canada

**Keywords:** Developmental Disability, Family Support, Natural Support, Formal Support

## Abstract

Evidence suggests that integrated support, combining both natural and formal supports, is often essential for individuals with developmental disabilities to achieve their preferred quality of life. However, studies are limited on how to organize supports so that people with developmental disabilities and their families find a balance between formal and natural supports. Often, there are systemic and personal boundaries around the nature and extent of support that can be offered to persons with developmental disabilities through formal mechanisms, yet the value of natural supports in the lives of persons with developmental disabilities is often undervalued in society. Therefore, the objectives of this study were to explore formal support providers’ perspectives on (a) the unique skillsets and attributes of natural support providers and formal support providers; and (b) how we might best enable both natural and formal supports for persons with developmental disabilities and their families. Following a qualitative approach, we interviewed 16 formal support providers working with adults with developmental disabilities and their families via Zoom. We analyzed data using thematic analysis. We organized results into three themes: the role of natural supports, the role of formal supports, and strategies to best configure a system of supports. Results imply that there is a need for investment of funding to incentivize both support structures for adults with developmental disabilities and their families. Future studies should explore the perspectives from people with developmental disabilities and their natural support providers.

Individuals with disabilities contribute to a significant portion of the Canadian population. Based on information from the Canadian Survey on Disability of 2017, 22% of the Canadian population aged 15 years and over (about 6.2 million) have one or more disabilities, and nearly 5.1% (or 315,500) of Canadians in that age group reported that they have developmental disabilities (Morris et al., 2018). Families play a crucial role in supporting adults with developmental disabilities across the world, including Canada (Chadwick et al., 2013; Navas et al., 2021). It is estimated that 7.8 million Canadians aged 15 and older are involved in providing care to their family members for various reasons, such as long-term health conditions, a physical or mental disability, or problems related to aging. Of the 7.8 million caregivers, 8% (over 600,000) provided care to their child with a long-term health condition or a physical or mental disability (Statistics Canada, 2020).

Having a family member with a disability is rewarding, as well as challenging if appropriate supports are not available (Chadwick et al., 2013; Nurullah, 2013; Sheldon et al., 2021; Yoong & Koritsas, 2012). People with developmental disabilities and their families need a broad range of support to cope with disability. Some of the commonly reported support needs are informational, emotional, and instrumental support (Friesen et al., 2010; Shooshtari et al., 2012). Instrumental support entails practical assistance or advice concerning strategies and assistance in problem-solving (King et al., 2006). Families also often need financial support (e.g., for assistive devices and cost of disability-related care) and assistance navigating the disability system (Friesen et al., 2010). Adults with disability also need support to facilitate their participation in education and the labour market, such as mentorship, work training and accommodation (Jetha et al., 2019; Lindsay et al., 2016; Petner-Arrey et al., 2016). The support needs of an adult with a disability could vary across the lifespan because of the developmental transition in human life (Hole et al., 2013; Nguyen et al., 2018; Shooshtari et al., 2012). Needs can also vary based on the types and severity of disability (Ball et al., 2012). Some people with developmental disabilities may need lifelong support in many areas of life, such as activities of daily living, housing, employment, education, and transportation (Shooshtari et al., 2012).

It is sometimes important to understand disability-related supports based on particular classifications, such as if the support is provided formally or naturally. In this study, we defined formal supports as those provided through paid providers (e.g., personal support workers, social workers, psychologists, and educators) or government programs (e.g., Ontario Disability Support Program). We defined natural supports as being inclusive of all types of unpaid support derived from the combination of love, loyalty, and necessity (e.g., self-help/peer support). Currently, there is a lack of literature concerning how to create a balance between formal and natural support for Canadian adults with developmental disabilities and their families to address their needs (Jansen-Van Vuuren et al., Under review). Therefore, the objectives of this study were to explore service providers’ perspectives on (a) supports that are unique to the skillsets and attributes of natural support providers vs. formal support providers; and (b) how we might best enable both natural and formal supports for persons with developmental disabilities so that they might live a high-quality life of their choosing. Specifically, the research addresses the following two questions: what are some illustrations of support services that are distinctly aligned with the skill sets of formal support providers in contrast to the natural support providers? How can we design a system of support for persons with developmental disabilities that acknowledges the relationship between formal and natural supports while highlighting the significance of family and other natural support within their local community? This study is one piece of an overall research project that is also exploring the opinions and perspectives of adults with developmental disabilities and their families about how policies and organizations can support families to achieve their desired balance of formal and natural supports in disability.

## Literature Review

Researchers have examined the role of formal and natural support for people with developmental disabilities and their families (Casey & Stone, 2010; Tétreault et al., 2014). Studies found that better access to both kinds of support helped people with developmental disabilities to cope with disability and increased the level of independence and social participation (Friesen et al., 2010; Naganathan et al., 2016; Petner-Arrey et al., 2016; Rudman et al., 2016). Support also plays a significant role in improving the wellbeing and quality of life of individuals with developmental disabilities and their families (Brown et al., 2003; Canary, 2008; Casey & Stone, 2010; Naganathan et al., 2016). For instance, a study with students with developmental disabilities in five provinces in Canada found that informal support (e.g., encouragement and affirmation) from family, friends, and university staff increased individuals’ sense of belonging and involvement in social interactions and self-esteem in reaching their career aspirations (Berry & Domene, 2015). Studies with family members of children with developmental disabilities also reported similar outcomes (Canary, 2008; Nuri et al., 2020). For instance, in a review of the literature, authors found that families who received higher levels of informal social support from friends and family were likely to report lower parental stress, greater feelings of parental empowerment, and higher levels of marital satisfaction (Canary, 2008). In a cross-national study, authors found that having contact with other family members of children with developmental disabilities was beneficial for effective life management within the family (Wilgosh et al., 2004).

A study in Ontario found that informal support from parents and social networks was important for adults with developmental disabilities in securing and sustaining employment (Petner-Arrey et al., 2016). Parents and family friends were specifically helpful to adults with intellectual disability in securing job placement and volunteer opportunities. In some instances, parents helped their children with developmental disabilities understand job expectations and provided on-the-job assistance that helped people with intellectual disabilities to sustain work over time (Petner-Arrey et al., 2016). In another study, it was found that hands-on support from families helped farmers with disabilities return to work (Friesen et al., 2010). Likewise, Khan et al. (2021) found that practical assistance (e.g., driving and/or accompanying to appointments, communicating with care providers, and purchasing baby supplies) from family and friends positively shaped the perinatal care experiences of women with intellectual disabilities (Khan et al., 2021). A study with older adults found that people aging with significant long-term impairment felt better when they were able to share their thoughts, feelings, and problems with others (Casey & Stone, 2010).

Studies also revealed formal support by professionals and its benefits for individuals with developmental disabilities and their families (Heifetz et al., 2019; Khan et al., 2021). For instance, a study of mothers with intellectual and developmental disabilities reported that practical help in completing paperwork from paid service providers (e.g., parenting therapists, social workers, nurses, and physicians) helped them reduce stress and improve mental health (Heifetz et al., 2019). In a Canadian study, individuals with developmental disabilities reported that formal support (e.g., budgeting, helping to establish routines, and completing paperwork to access resources) from nurses and disability-related care workers helped them to navigate the disability support system (Khan et al., 2021). Further, studies examined formal support and family wellbeing. Some studies found that families of children with developmental disabilities felt greater wellbeing and empowerment when services are family-centred (e.g., providing needed information and treating parents with respect) and addressed family needs (Davis & Gavidia-Payne, 2009; Dempsey et al., 2001; Dunst et al., 2007). Another study of older adults with multi-morbidity in Canada found that formal support, such as personal support workers, eased the caregiver burden by providing support in activities of daily living. It also helped to improve relationships between the caregiver and care-receiver and reduce negative emotions (Naganathan et al., 2016). Similarly, respite care relieved families from stress and fatigue caused by responsibilities related to their family members with developmental disabilities (Tétreault et al., 2014).

It is evident that both formal (e.g., paid service providers or government support systems) and natural/informal support (e.g., family, friends or neighbours) are essential for adults with developmental disabilities and their families. However, people with developmental disabilities and their families often struggle to find a balance between formal and informal support. Individuals with developmental disabilities and their families sometimes find that formal support is limited, difficult to access in a timely manner and/or not inclusive to meet the unique needs of individuals with developmental disabilities (Chouinard & Crooks, 2005; Hole et al., 2013; Lunsky et al., 2007; Shooshtari et al., 2012). Therefore, many people with developmental disabilities primarily rely on support from their informal sources, which tends to increase the caregiver burden and risk of caregiver burnout (Milic Babic & Dowling, 2015; Naganathan et al., 2016). Ineese-Nash et al. (2018) further demonstrate that the current Canadian system operates from a colonial framework that does not align with Indigenous ways of child-rearing and knowledge of human diversity. The underlying ideological differences can lead to conflict for Indigenous families as they seek to maintain their natural, cultural understandings of development and support while accessing formal support for their children. Oftentimes, people with developmental disabilities and their families are not able to access natural support, given the values around which our system is structured. The voice and perspectives of formal support providers are often seen as the priority and the authority, and natural support providers are diminished, sidelined, or excluded from spaces where their contributions are critical and often irreplaceable by the formal support system (Biason, 2022; Reynolds et al., 2018). It is critical to further explore the contributions of both types of supports and create a system that fosters and enables them to work in tandem as per the needs and desires of the support users.

## Methods

### Positionality

The research team members came from diverse backgrounds, which enabled us to use multiple lenses to explore this topic in detail and interpret results with more nuances. For instance, four research team members have extensive experience working with adults with developmental disabilities in Canada and globally. Further, there were two family members of adults with developmental disabilities and a representative of an organization that provides supports to adults with developmental disabilities on the research team. These individuals guided the identification of the research questions for this study and were involved from the beginning to the end of the project. They brought insider perspectives, with experiences providing (or accessing) both natural and formal supports. Engagement of these colleagues with lived experiences of disability-related supports in this inquiry shaped our interview guide and made it easier for us to gain access and recruit study participants. Their insider perspectives also helped us plan for diverse avenues to share the research findings for a wider impact, such as preparing a lay summary for adults with developmental disabilities and their families and sharing the study outcomes with different organizations and policymakers working with people with developmental disabilities.

### Study Design

We conducted this qualitative descriptive study (Sandelowski, 2000) with formal support providers who were selected purposefully across different provinces of Canada. Qualitative descriptive study is a method that generates data that describe ‘who, what, and where of events or experiences’ from a subjective perspective (Kim et al., 2017). In choosing this approach, our goal was to provide straightforward descriptions of perceptions and reveal actionable information with direct implications for practitioners and policymakers (Neergaard et al., 2009; Sandelowski, 2000).

### Study Participants and Recruitment

Study participants were formal support providers who (a) had at least three months of experience working with adults with developmental disabilities and their families in Canada as paid, formal support persons and (b) were willing to have a virtual interview. We excluded service providers who (a) did not have at least three months experience working with adults with developmental disabilities; (b) were not based in Canada; (c) did not provide supports to persons with developmental disabilities specifically; or (d) primarily offered supports in an unpaid role, as a natural support provider or volunteer. We opted to exclude them because we thought if service providers do not have minimum experience working with individuals with developmental disabilities as paid service providers, they might not be able to share rich and relevant information related to our study.

We followed three steps to recruit participants. First, the research advisory group/team members (people with developmental disabilities and family members of people with developmental disabilities, and representatives from support organizations) identified organizations that provided support to adults with developmental disabilities in Canada and key contact persons from those organizations. They also gave feedback on the importance of capturing a diversity of types of formal support providers (e.g., recreation, housing, mental health supports, direct support workers, and clinical professionals). Second, based on that contact list, the research coordinator made the initial contact with the representatives of the organizations via email or phone call and asked for help with identifying potential participants from their organizations. Third, based on recruitment rates (i.e., low response rate), the research coordinator searched for community organizations across Canada that provided support to individuals with developmental disabilities. The research coordinator also shared the participant recruitment flyer with clear instructions about eligibility criteria with the organization’s representative and requested them to share the flyer widely within their organizational networks. Individuals who expressed interest after reading the study flyer were recruited and connected with the interviewer.

### Interview Guide Development

We used a semi-structured interview guide to conduct interviews with participants (see Table 1). The interviewer also asked probing questions to elicit detailed information about the study objectives. All authors were involved in developing the interview guide, which was informed by the literature review and research underway within the wider research project umbrella. We obtained feedback on the interview guide from advisory committee members (e.g., family members of adults with developmental disabilities, disability advocates, formal support providers, and community partners working with adults with developmental disabilities) prior to finalizing it. The person conducted the interviews had significant experience in conducting semi-structured interviews, both in person and on Zoom, and adjusted question phrasing and order to best accommodate the flow of the conversation. Given our knowledge of the formal service sector and current levels of burnout and shortages (offering likely challenges for study recruitment), as well as the insight gained through consultations with families, people with developmental disabilities, and organizations that serve them through our study advisory board, we did not believe it was necessary to formally pilot the interviews before commencing the study.

**Table 1:** Interview guide

1. Please introduce yourself – what is your job and how do you support persons with developmental disabilities and their families? 2. How would you define the term “natural support”? 3. How do you interact with the natural support network of your client with a disability? 4. What do you think that friends, family, and community do for a person with a developmental disability that paid people like yourself cannot? 5. On the other side, what can paid people do that friends, family, and community can’t? 6. Sometimes you might want to do more to support a person with developmental disabilities, but you might be unable to, due to the system or the way your role is structured or for other reasons. Could you tell me about any times you have experienced a tension with your job where there were boundaries around the way you support and interact with your clients? 7. On the other hand, we have heard from persons with disabilities and families that sometimes, people who start out as formal supports become a part of their natural support network. Do you have experiences you might be able to share where you have become part of someone’s natural support network, offering support to them outside of your paid role, out of a sense of love, loyalty, or necessity? 8. How do you navigate a situation when the line between natural and formal support becomes difficult, or there is a misunderstanding of what the boundaries are? 9. How do you foster natural connections through your work? 10. If you had a magic wand, what might you do to improve systems of support for persons with developmental disabilities in Canada? 11. Is there anything else you would like to share with us today that might help us to understand how we create systems that best support individuals and families to find the balance of formal and natural support that works best for them?	

### Data Collection and Analysis Process

One of the authors, who was trained and experienced in conducting qualitative interviews virtually, conducted interviews via Zoom. All interviews were conducted in English and on a mutually agreed-upon date and time. With the participants’ verbal consent, all interviews were audio and video recorded. We kept the audio recording and discarded the video. The interviewer also took field notes during and after the interview. In the field notes, the interviewer wrote her impression about the interview (e.g., what went well and what did not go well), participants’ facial expressions or any word that was given emphasis. The field note was shared with the senior author for her guidance on how to improve subsequent interviews. We provided $25 via e-transfer to each participant to compensate for their time. This study was approved by Queen’s University General Research Ethics Board (application number: 6032728).

We hired a professional transcriptionist to transcribe all interviews verbatim, which was checked by the interviewer for accuracy. All identifiable information was removed from the transcripts before analyzing the data. We followed inductive thematic analysis. The process of thematic analysis involved reading and re-reading the transcript, open coding, merging coding based on similarities and differences and collapsing coding into themes or sub-themes (Braun & Clarke, 2019). Two authors reviewed all the transcripts independently and engaged in open coding. During initial coding, the coders developed a detailed description of the codes that was revisited, added to, and refined as more transcripts were read. Each coder merged their codes into sub-themes based on similarities. After completing the data analysis, two coders shared codes and sub-themes with the senior author to compare and contrast both sets of analyses. The two coders and the senior author met multiple times, compared codes and reconciled all coding discrepancies through discussion. The senior author read all the transcripts prior to the meeting, which helped us negotiate the coding discrepancies. The coders and the senior author also discussed sub-themes, came to a consensus on sub-themes, and identified themes for each research question. All authors reviewed and agreed upon the final themes and sub-themes.

### Credibility and Trustworthiness

We ensured credibility and trustworthiness by employing several measures outlined by (Brantlinger et al., 2005). First, more than one author was involved in data analysis, which enabled us to minimize biases and validate themes as they were developed. We also obtained feedback on themes and sub-themes from all authors, three of whom work within formal support organizations. Second, we jointly reflected on our own positionality and how that might factor into our analysis. This study is an outcome of collaborative work where the researcher, family members of adults with developmental disabilities and community partners were involved from the beginning to the end of the project, which helped us to explore the phenomenon using multiple lenses. Third, we kept an audit trail of decisions made during the data collection and analysis. Fourth, the researchers were involved in peer debriefing during the data collection and analysis and provided critical feedback on methods, analyses and interpretations of study findings. We member-checked study findings with the project advisory board and at a large conference of formal developmental service providers in Ontario.

## Results

### Participant Demographics

We interviewed 16 service providers from four provinces of Canada, of which British Columbia and Ontario were the most frequent. Of the 16 participants, 62.5% (*n* = 10) were female, and 31.3% (*n* = 5) were male. One participant did not disclose gender identity. Most participants (*n* = 10) had over twenty years of experience working with adults with developmental disabilities. A majority (62.5%, *n* = 10) of them had post-secondary certificates or degrees (Table 2).

**Table 2:** Participant demographics

Category	Number (%)
Gender	
Female	10 (62.5)
Male	5 (31.3)
Decided not to disclose	1 (6.2)
Number of years of experience	
< 5 years	4 (25.0)
5 − 10 years	1 (6.2)
11—20 years	1 (6.2)
> 20 years	10 (62.5)
Provinces	
British Columbia	7 (43.7)
Ontario	7 (43.7)
Nova Scotia	1 (6.2)
New Brunswick	1 (6.2)
Education	
Secondary School Diploma	1 (6.2)
Post Secondary Certificate Diploma/Degree	10 (62.5)
Post graduate	5 (31.3)
Occupation	
Director or equivalent	4 (25.0)
Manager/Team Leader	3 (18.7)
Social/Community Inclusion Coordinator	2 (12.5)
Personal/Developmental Support Worker	2 (12.5)
Independent Facilitator	1 (6.2)
Inspirational Speaker	1 (6.2)
Sexual Health Educator	1 (6.2)
Respite Services Supervisor​	1 (6.2)
Youth Development Counsellor	1 (6.2)

### Qualitative Findings

Participants shared information about the role of natural and formal supports for adults with developmental disabilities and how we can configure the system of care that incentivizes both natural and formal supports. We have organized findings into three themes and nine sub-themes (Table 3).

**Table 3:** Themes and sub-themes at a glance

Themes	Sub-themes	Examples of codes
Theme 1: Role of natural support	• Love and sense of belonging	Unconditional love, sense of belonging, security, stability, hold history, comfort, long-lasting relations, stay forever, deep connection, intimacy, genuine connection, longevity, sense of community, quality time, and hugs
	• Advocacy, employment and future planning	Advocacy, finding employment, setting up a pension plan, and talking about disability rights
Theme 2: Role of formal support	• Provide specific expertise	Treatment, counselling, consultancy, accessibility measures, expert opinion, filling out the application form, helping navigate the system, and sharing information
	• Enable natural support to flourish	Respite care, role clarification and the reciprocal nature of the relationship, help families establish a connection, assist in writing emails, and help find like-minded people
	• Assist in activities of daily living	Cooking, cleaning, bathing, and shopping
	• Limitations of formal support	Professional boundary, liability, organizational rules and regulations, shortage of manpower, lack of funding, and strict eligibility criteria for certain services
Theme 3: Strategies to configure the system of supports	• Facilitate community living	Community living, reciprocal relationships, more eyes, a safe environment, surrounded by friends, disability awareness, stigma and discrimination
	• Incentivize both natural and formal support provisions	More resources, flexibility to use government funds, trained disability service providers, sexual health educators, developmental therapists, and liveable wage for disability service providers
	• Reduce barriers to accessing formal support	Paperwork and fragmented services

#### Theme 1: Role of Natural Support

##### Love and Sense of Belonging

Almost all participants (*n* = 15) discussed the critical role of natural support for adults with developmental disabilities. Participants indicated that natural supports, specifically from family, friends and community, provide safety to a person with developmental disability, as well as a sense of belonging, love, security and stability. Natural support can also create an enabling space for adults with developmental disabilities to share their feelings. Participants noted that the depth of the relationship and emotional connection found within natural support is enormous, which may not come from formal sources. Natural support can also bring joy to an individual with a disability, which is critical for wellbeing and quality of life:

That’s not enough for someone’s quality of life to have a service provider as the only support. They need their own networks. They need opportunities to have a role in their families, to contribute and to be an adult with their aging parents. [Participant 14]

If they [persons with developmental disabilities] have visitors who are their immediate families, their sibling or their friends, it’s a nice day for [my] clients. They talk, they laugh, they feel better on that day. I feel it is very important. This is something which, even if, as a developmental support worker, we are there literally every day with our clients, but I feel we have a boundary. The client feels a more emotional connection with their natural support than with us. I feel it eases them. [Participant 11]

Participants also noted that natural support from immediate family and community is more likely to be sustainable and available in any adverse situation when formal support cannot function. Participants recounted how the natural support from family and church community of their clients with developmental disabilities assisted their clients during the COVID-19 pandemic:

One of our clients [with a disability] lives alone by herself in an apartment. She had to go in for surgery. When she was discharged, she couldn’t move around in her apartment. It’s hard to get up and make her meals or to clean up after her surgery. Between her family and her church and her friendship, they had a whole two weeks organized in terms of this person dropping off meals, this person is going along and cleaning her apartment. Family was calling her every day, like someone scheduled for the morning. They had a whole plan going. Then we’d go along as staff and still be able to check in, the regular thing, and do our four hours of support. [Participant 10]

##### Advocacy, Employment, and Future Planning

Participants (*n* = 7) reported that natural support is incredible for advocating for system change, gaining access to employment and achieving long-term financial goals. Participants recounted natural support, especially that from family, helped their clients with developmental disabilities find jobs and set up retirement saving plans. One participant noted how families can advocate in a different way for persons with disabilities than formal support providers can.

They [family members] provide a different level of advocacy, I’m going to say, because coming from a paid position, it is always difficult to be 100% neutral in that situation. As much as we do like to say this is 100% what we’re trying to do, sometimes, whether you realize it or not, we are working with the bias of our work environment in terms of trying to support them to fit into what works for our organization, whereas that unpaid support person is 100% truly coming on behalf of that loved one. They’re not looking at the financial constraints of an organization or the environmental constraints of that. They’re looking at their loved ones and trying to support and advocate for the best thing for them. That really is, to me, what loved ones and natural support can bring as opposed to a paid support worker. [Participant 14]

#### Theme 2: Role of Formal Support

Similar to natural support, formal supports can often play a significant role in many aspects of the lives of adults with developmental disabilities and their families. The contributions of formal support have been organized into four sub-themes: (a) provide specific expertise, (b) enable natural support to flourish, (c) assist in activities of daily living, and (d) limitations of formal support providers.

##### Provide Specific Expertise

Participants (*n* = 10) reported that formal support can provide expert-level technical advice using their specific education, training, and on-the-job experience. They indicated that formal support providers have the expertise to know the ins and outs of systems navigation by virtue of doing it for many people as part of their job. For example, some support providers indicated they help individuals and families navigate seemingly impossible systems and paperwork processes that enable them to access other formal supports. Families may not know what they can access or how exactly to access it, whereas formal support providers better understand the intricacies and mysteries of systems and paperwork navigation, having navigated these systems many times for others. Other support providers indicated they have specific expertise in behaviour management or accessibility that they are able to bring to individuals and families.

They [family] can’t handle their [individual with a disability] behaviours, they can’t handle the individual when they get upset. They’re hitting the aging family member and they don’t know how to handle that. They can’t do that. They’re getting beat up every day or they’re emotional, they can’t handle this physically, they can’t handle it. We do have access to some of our services, like behavioural consultation, that help staff are trained and have experience in working with people with challenging behaviours and developmental disabilities. [Participant 10]

I am working very closely with organizations … [name of organizations] to raise awareness on the importance of accessibility and universal design practices. I know how to read blueprints, talk to building designers and architects about building spaces to gold standard, which goes above and beyond building code. I also talk about accessibility and human resource policies when it comes to hiring people with developmental disabilities. The best practices for employment strategies for persons with developmental disabilities. [Participant 5]

##### Enable Natural Support to Flourish

Participants (*n* = 12) reported that formal supports play a significant role in enabling natural support to flourish. They said formal support assisted adults with developmental disabilities in setting boundaries, building skills and knowledge to recognize the reciprocity in natural support relationships, and creating opportunities for a person to get out in the community and make natural connections with other people. Two participants recounted:

A family can’t be everything to their child or their loved one. It’s, I think, healthy for a loved one to want a little bit of autonomy, especially when they become young adults. I have a son who also lives with a disability, and he doesn’t tell me everything. He doesn’t want me to know everything, and I don’t want to know everything. I want to know that he’s happy, he’s healthy, he’s connected, he’s doing meaningful things in his world, things that he wants to do, and he’s developing and he’s growing. [Participant 13]

We’ve supported people to think about their role …thinking about what it means to be an aunt or an uncle if you have siblings who are starting families of their own and helping people to think through. Even things like getting a birthday card for your family members and reaching out to them to offer to host a birthday or a Christmas dinner or something for your family. That reciprocal nature of relationships so you’re not just on the receiving end and expecting other people to give to you. [Participant 2]

Participants reported that establishing connections with community members is essential for adults with developmental disabilities to alleviate loneliness, especially when the close family members of adults with developmental disabilities are no longer around. It is also critical to improve the sense of belonging and, thus, a better quality of life. Formal support offers a range of activities that facilitate interaction between adults with developmental disabilities and community members. They provide one-to-one support to their clients with developmental disabilities to establish meaningful and authentic connections with the community through email, phone conversation or social media. Some formal support providers have taken innovative approaches to foster natural support of individuals with developmental disabilities. One participant recounted how some formal support providers connected individuals with developmental disabilities with like-minded people in their community:

When we’re thinking about getting people connected to people and having connections, and potentially then being natural support, would be finding what they find is valuable. A mom recently was like, “Oh, well, he should volunteer here, or there, or wherever,” but asking what the purpose of that would be and finding out where that person actually want, what they find is valuable. In this case, it was they actually had an interest in photography. You might look at finding if there’s either natural support in their life that enjoys photography that they can connect with, or if there’s a group out there that person can join to practice photography or lend their photography skills to volunteer with an organization, and then maybe meet people where they can receive support. [Participant 12]

When we get a family referred to us who needs a break, a respite from caring for their child, that’s usually the first place we’ll start. Is there anyone in your circle or your network? Is there a neighbour? Is there someone on your street? Do you have a niece or a nephew? Because often that’s comforting for the family or reassuring to know. Sometimes it backfires as well, but on balance and on principle, that’s the case. I find though that as folks age, it becomes, for better or for worse, more difficult for families to identify folks in their circle. [Participant 7]

##### Assist in Activities of Daily Living

Participants (*n* = 6) reported that formal support providers offer a range of practical support in day-to-day activities for adults with developmental disabilities. These include but are not limited to cleaning, cooking, and bathing. Participants also highlighted the role of formal support providers in providing nursing care to those who have high care needs. Participants noted that natural supports may find it challenging to provide 24/7 care to adults with complex medical needs, but formal support can offer multiple people in multiple shifts to ensure that person is supported 24/7. It also gives the natural support providers a break and time to recharge and refresh. However, finding 24/7 care with multiple shifts could be challenging for families with limited financial support and could potentially lead to further stress. Participants indicated the need for families to have financial support from the government and incentivized care for adults with developmental disabilities to ensure access to continued and sustained formal support when natural support is not present.

Sometimes, natural support cannot be present 24/7 with our clients [with high care needs]. That is one reason why we have this whole developmental support worker 24/7 with the clients. Giving time, shopping with them, showering them, health and hygiene, and preparing food on a daily basis. [Participant 11]

##### Limitations of Formal Supports

Although formal supports are instrumental in providing care and services to adults with developmental disabilities, participants (*n* = 7) reported some limitations that affected their ability to provide optimal support to adults with developmental disabilities. Resource scarcity, professional boundaries and liability were some major constraints preventing formal support providers from going the extra mile for their clients with developmental disabilities. Participants reported that a paid service provider has to be mindful of policies, procedures, and boundaries when they work with individuals with developmental disabilities. Further, a paid service provider is often contacted for certain hours and days of the week for specific tasks, which is insufficient to meet their clients’ needs. A number of participants recounted how resource constraints, professional boundaries and liability deterred them from doing more for their clients with developmental disabilities:

One of our staff wanted to join our client for an exhibition that cost only $12, but then because it’s an out-of-town assignment, collective agreement says that we have to go along and pay out an additional eight hours per day for each 24 h period. Even calculating those extra because it’s three days away, so extra 24 h, like 8 h times three, 44 h. Having to give that to our payroll person, asking them to calculate that cost plus other benefits, bits of things like that. Even those 24 h amount to an extra $1,000 plus on top of that. It’s a $12 exhibition that he wants to go to and the trip costs $2,000. Half of that is just additional staff wages. Though the staff had gone along and said, “Well, can I just go along and just don’t pay me that? I want to just take them. I just do my eight hours and you pay me for that, but I don’t want the extra eight hours per day that’s costing the individual next to $1,000 just for that. I feel bad about that.” I said, “No.” I said, “I’m sorry, we can’t do that.” [Participants 10].

#### Theme 3: Strategies to Configure the System of Supports

Participants shared a number of strategies that can help us understand how we might shape a system of care for adults with developmental disabilities that recognizes the interdependence of formal and natural support and the central role played by family and other natural supports available in the community.

##### Facilitate Community Living

Participants (*n* = 9) emphasized the importance of community living for adults with developmental disabilities and suggested organizing both formal and natural support around them by taking into consideration individuals’ strengths, culture and priority needs. Participants specifically noted that if adults with disabilities can live in their home community, close to family and friends, while receiving the formal support they need and maintaining autonomy, it would be the best strategy instead of putting them in formal long-term care facilities or traditional group homes. Two formal support providers, one of whom was also a mother of an adult with a developmental disability, described how the existing system discourages community living for adults with disabilities:

If we want to build a one-bedroom apartment onto our little house or put a granny flat in the back either for our daughter or for a 24/7 support person to live to provide support for her and a roommate in our house, there’s no funding for that. Even though it would be a lot cheaper than having her in a group home or long-term care. [Participant 1]

If you moved into a group home, it was often very difficult for people to get out of that. Your funding was attached to the facility. You had staff that were shared with the other people in the house. Although you had the freedom to leave if you wanted to, there was no guarantee that you would have funding to support you to do something different. [Participant 2]

Participants mentioned that when people with developmental disabilities live in the community and are surrounded by family, friends and other natural supports, they can build a reciprocal relationship with their community, which could give them the best life experiences. However, overreliance on natural support while facilitating community living could potentially put people with developmental disabilities in vulnerable situations if formal support is insufficient. A number of participants recounted:

Systems will not result in people having a good life. Friends, and family, and connection, and contribution, and reciprocity are the elements of a good life. A system can play a role in facilitating that…Things are best when they lean more toward natural and formal. To the extent that people can have a good life in a community with minimal access to paid support would be the ideal, but not a loss of paid support such that you couldn’t have a good life. It’s a balance because I know that there are some models I’ve witnessed where there’s a really strong emphasis on natural supports, but the risk is that you over-demand and they start to pull away, and then the person is left even more vulnerable because they don’t have sufficient formal supports. [Participant 7]

Most of the young people who are referred to us are Indigenous and almost all of them grew up in care and that has lifelong implications. If you take a child out of their home and place them in foster care, you’re creating trauma and probably adding to trauma that was already there. Anything we can do to support Indigenous communities, to take care of their children, and to raise healthy, functional adults who know their culture I think would be a very worthwhile investment. [Participant 2]

Participants recognized that for some adults with developmental disabilities, group homes might be the only option for living, especially for those who do not have strong family connections and social networks, such as older adults. However, participants suggested that an improvement is needed in the group home structure so that people with developmental disabilities who have to live in those settings still enjoy life.

A number of participants (*n* = 7) reported that stigma and discrimination against people with developmental disabilities are still prevalent in Canada, which could be a barrier for adults with developmental disabilities living in the community. Therefore, participants emphasized the need to have ongoing conversations about disability. Participants highlighted that school could be a starting place for awareness rising because it could facilitate children learning about disability at a young age.

I think if there was education around this population [people with developmental disabilities] as well as the ability to communicate more freely, I think people would not be as fearful to engage with individuals with developmental disabilities…. To be able to break down some of those walls and to see some education around how to engage well with those individuals would be amazing. [Participant 14]

##### Incentivize Both Natural and Formal Support Provisions

Participants highlighted the need to incentivize both natural and formal support provisions to improve the system of care for adults with developmental disabilities and their families. Participants underscored that more financial supports, such as tax breaks for families who want to provide support to their loved ones with developmental disabilities, could be one of the strategies to incentivize natural support. Additionally, participants discussed the necessity for respite care to support family members of adults with developmental disabilities. Therefore, they highlighted the importance of increased financial support for organizations to provide such services for families of adults with developmental disabilities. Participants stated that both systems need to co-exist and support one another better.

I think that a lot of times there’s a struggle for friends and family members who want to be there for the individual in their life who has a challenge, but they just can’t afford to do that because they have to make an income. If there were more subsidies available for those families, or even tax breaks that were available, so that they could provide those supports, and do so without having to worry about how they’re going to put food on the table, I think that would make a big difference, especially now because people are having hard economic times. [Participant 5]

More funding for organizations that can provide respite visits for people who have developmental disabilities because families that very much want to support people who have disabilities, like their children or their relatives of any sort, sometimes they need a break. Sometimes they need to focus on their children who don’t have developmental disabilities. They need to be able to have one night without screaming or upset or things like that. Organizations where this person can go and stay and have a little bit of a vacation, something along those lines, that would be just phenomenal because I think it goes a long way in saving the sanity of people who support people with developmental disabilities. [Participant 15]

Nearly all participants (*n* = 13) highlighted the need to allocate more financial and human resources to improve support structures for adults with developmental disabilities. Participants stated that these resources need to be allocated both at the individual and system levels. At the individual level, participants stressed the importance of providing improved financial support for adults with developmental disabilities so that they could live more comfortably and access more services. Participants reported that the amount of financial support each individual with a disability receives through a formal disability support scheme does nearly nothing to meet the needs of persons with developmental disabilities. As a result, adults with developmental disabilities skip many services that are essential for their development and community integration. Participants believed that additional financial support would enable adults with developmental disabilities to live in their community and participate in activities that are important for their mental health and wellbeing, such as recreational activities.

I would say because a lot of our clients, there isn’t a lot of money. The amount they [adults with developmental disabilities] get for their disability is barely enough to cover rent, let alone accessing social groups. It always becomes this challenge of it’s not a matter of not wanting to provide that support. It’s a matter of I have to pay rent and to keep the lights on and I have to pay my staff. That is all very, very expensive. [Participant 4]

Participants also reported that individuals with developmental disabilities should be given more choices to spend the financial support they receive from the government under different schemes. It would enable adults with developmental disabilities to direct their funds based on their priority needs for a given time. A participant noted:

I think it’s really important for people to have their own funding that they can make their own choices. Individualized funding for people with some support around how to use that money. For example, if someone’s parent dies, they might choose to use more funding for some therapy and counselling, and grief work this year. Then next year, they might want to use the money to help establish an apartment or something. [Participant 1]

In the province of Ontario, my understanding is that the province would be ready to put out about $200 a day to support my daughter in a group home, but they’re not going to put a penny towards her providing support for her in her own home as she ages. They will put a penny toward, but her funding is a fraction of what that would be. If she had the choices around what was really important to her, how would she use her money? How would her micro board help her to make financial choices around the things that were important to her? I think there are many, many people who need more choice around how their money is spent. [Participant 1]

At the system level, participants emphasized that formal support agencies working with adults with developmental disabilities and families should have more access to funds as well. This will enable the formal support providers to hire more people and introduce more programs and services that are needed for adults with disabilities and their families. For instance,

participants noted that services like sexual health education, assistive technology or music therapy are underfunded. Families have to pay from their pocket to avail of these services if they do not have insurance, which in most cases is not an option for many adults with developmental disabilities and their families due to financial hardship.

We have a really good medical system here in Canada where a lot of things are covered, but sometimes certain specialized programs or specialized equipment they’re not covered. It’s because they’re seen as not necessarily required for that person on a medical basis, or sometimes they only are able to receive coverage from insurance up to a certain amount, but if they had that piece of equipment or that life-changing tool, it would change their life dramatically in terms of being able to socially interact or get out there. [Participant 5]

If we’re wanting to do different stuff or innovate in any way or create new development or something, or even research certain things that will, in effect, have some benefit back to our individuals or something, then there’s no money, there’s no budget or anything like that. There isn’t that flex to do that. [Participant 10]

Participants also highlighted the need to educate and fund more skilled support positions, such as doctors, nurses, developmental support workers and mental health support workers. Participants reported that there is a shortage of these professionals, contributing to long wait times, which harm the health and wellbeing of adults with developmental disabilities. Additionally, participants reported that the government needs to invest more resources in people who are working in the disability field. Participants specifically indicated that typically, formal support providers’ salaries are very low, which forces people to leave the profession and thus puts pressure on natural supports. They indicated that they believe government should increase salaries to attract more people in the disability field. Similarly, resources need to be allocated for pre- and in-service training for disability support workers.

We have a specialized mental health team for people with developmental disabilities in British Columbia. Typically, the only time you’re referring to that is if a mental health crisis is happening. Well, that’s a 6 to 12-month waitlist. Well, how responsive is that? The same as for behaviour support, a huge waitlist. It’s not to say that everybody needs those supports, but those are often some of the challenges folks face that they need support on in order for them to be able to access the community and be part of a community and be involved in the community without some of those stigmas and those other pieces attached to them. [Participant 8]

We fought for the wages we have now. I’m only now making a comfortable living wage. I’ve been in this field for a long time and now I’m making a wage where I’m not necessarily paycheck to paycheck every week. I’m also older, my kids are grown, but we don’t make that much money. I think the government seeing the value in the work that we do and supporting that. We’re an accredited agency. That’s an important piece. [Participant 13]

##### Reduce Barriers to Accessing Formal Support

Participants (*n* = 5) reported that adults with developmental disabilities experience barriers while accessing formal support. Participants specifically noted that adults with developmental disabilities and their families have to complete lots of paperwork to access formal support, which could be frustrating and tiring. Participants noted that existing services are not well connected, which may be the contributing factor to the access paperwork. Therefore, participants suggested that formal support provisions need to be innovative to minimize paperwork.

If you were a person with a disability, you shouldn’t have to prove it over and over again every level of application. To get your DTC [Disability Tax Credit] it’s like a five-page application that’s filled in by a doctor that often has a $50 fee. Not everybody has access to a doctor. If it’s proven one time that you have a disability, why should a doctor have to prove it again? [Participant 12]

If there is a way that people with a disability could automatically have access to the things that they should be guaranteed like the disability tax credit and a [Registered Disability Savings Plan^1^] even automatically set up. I think things with people with a disability should be more automatic and simple. There should be a lot more interconnection. [Participant 12]

## Discussion

This study aimed to explore service providers’ perspectives on (a) the unique contributions of natural support providers and formal support providers; and (b) how we might best enable both natural and formal supports for persons with developmental disabilities so that they might live a high-quality life of their choosing. Our study revealed several noteworthy findings that contributed to the limited research on the role of formal and natural supports for adults with disabilities. First, this study contributes to our understanding of different support systems (e.g., natural and formal) that are critical for people with developmental disabilities. Second, the findings shed light on potential avenues on how to organize services and resources so that people with developmental disabilities can remain in their community and feel supported. Third, the research outlines some research gaps that need to be explored to fill the gap in the literature. For instance, the applicability of the circle of support model in the Canadian context.

Consistent with previous studies (Giesbers et al., 2022; Sanderson & Aquino, 2023), participants of this study highlighted the unique role of natural support for adults with developmental disabilities. Participants noted that natural support can offer unconditional love and a sense of belonging, which are important to make life more meaningful (Ballin & Balandin, 2007; Friedman, 2023). As such, it is essential to nurture the natural support network of adults with developmental disabilities, and it should be the ultimate goal to facilitate community integration for people with developmental disabilities and their families. However, it is important to highlight that many adults with developmental disabilities find it difficult to engage in the community because they may not have enough connections in the community or they may feel hesitation about asking for help (Duggan & Linehan, 2013; Sanderson & Aquino, 2023). Formal support providers, such as social workers and facilitators, could help adults with developmental disabilities and their families to establish and maintain natural support networks in their community. Formal support providers can also work with natural support networks and help them develop skills that are needed to serve as effective support providers (Friedman, 2023). It may also be beneficial for the government to allocate more funding and enable flexibility and self-direction of existing funding to better enable natural support networks to more easily take on active support roles, if desired. Specifically, there could be greater consideration for tax breaks for natural supporters or application of shared living incentives typically reserved for shared living arrangements beyond the family also be extended to apply to family carers as well. Additionally, it could be beneficial for individuals with developmental disabilities who want to stay in their own home to have better access to funding to avail a range of formal support instead of losing such support because of their decision to reside in the community.

Similar to natural support, we found that formal support providers play important roles for adults with developmental disabilities in many aspects of their life, including activities of daily living (e.g., bathing, making meals, taking out for groceries, etc.). Support from formal sources in activities of daily living is not only essential for community integration but is also needed to enhance the quality of relationships between individuals with developmental disabilities and their natural support providers (Laragy et al., 2011). We also found that formal support providers can offer expert-level support in addressing disability-related issues, such as mental health support. However, resource scarcity and professional boundaries deter formal support providers from providing optimal care to their clients with developmental disabilities and their families. These limitations forced them to offer support only for a certain number of hours to address some specific needs without considering the holistic needs of individuals with developmental disabilities and their families. This could lead to mistrust between formal support providers and users and also hinder support quality (Topping et al., 2022). Open communication between formal support providers and users about the situation could potentially minimize misunderstanding. Future professional education might be provided to formal support providers to enable them to better connect with and engage natural support networks and other formal support systems in a person’s life to enable continuity and connection for the person receiving support, beyond support providers limiting themselves to knowing the person within the bounds of “our four hours of support”. Formal support providers could engage natural support providers and other members of the circle of care by organizing regular meetings, clarifying each other’s roles, and engaging everyone in goal setting based on the strengths and priority needs of adults with disabilities (Cress, 2015). This enhanced collaboration among all members of the circle of care is crucial for service satisfaction and the wellbeing of adults with developmental disabilities and their families. However, we understand these activities may require time and dedicated staff. It would be beneficial for support agencies to recruit, hire, and retain more formal support providers who have the skills to foster and enable natural support connections for adults with disabilities. Investment is needed to improve work conditions (e.g., increase salary) of formal support providers to attract people to work in the disability field.

Participants of our study also noted that disability-related funds should be allocated directly to individuals with a disability instead of allocating them to institutions. This will give adults with developmental disabilities and their families more control over their funds and the arrangement of formal support based on their priority needs (Laragy et al., 2011). This is important because the needs of adults with developmental disabilities are not static. It changes over time depending on one’s life circumstances. Therefore, our analysis strongly supports the idea of allocating disability funds directly to individuals with developmental disabilities as it has the potential to contribute to balancing the formal and natural supports for adults with developmental disabilities, especially when the resources are limited. Policymakers need to consider this recommendation when allocating funds for people with developmental disabilities and their families. Further, the government needs to allocate more funds to enable adults with developmental disabilities to participate in recreational activities. Future studies can explore how adults with developmental disabilities and their families want to see their funding arrangement to make their formal support a complement to their natural support.

Together, our findings indicate that adults with developmental disabilities and their families are better integrated when the formal supports interact with the natural supports. However, traditionally, formal and natural supports are viewed as two distinct systems with little interaction with each other, which may contribute to the high unmet needs of individuals with developmental disabilities and their families. Recent studies found that the interaction between these two types of support is becoming popular; therefore, authors of the earlier study suggested policymakers embrace the concept of integrated support rather than treating formal and natural support as separate entities (Reynolds et al., 2018; Sanderson et al., 2019). Reynolds and colleagues (2018) offer some insights into integrating formal and natural supports for individuals with developmental disabilities to achieve their preferred quality of life. According to the authors, formal supports can enhance natural supports by (a) recognizing and enhancing a person’s capacities, (b) strengthening and connecting social networks, (c) leveraging resources within environments accessed by all citizens, and (d) utilizing technological innovations (Reynolds et al., 2018). Our analysis also suggests that adopting these strategies in the Canadian context could potentially contribute to a more comprehensive and inclusive support system for adults with developmental disabilities. Future studies could explore the perspectives of individuals with developmental disabilities and their families on how to integrate these two support structures to meet their needs.

Almost all participants of our study recommended that adults with developmental disabilities will have a better life if they live in the community and are surrounded by family and friends. Evidence also suggests that deinstitutionalization of care and support enhances inclusion, interpersonal relationships and the wellbeing of people with developmental disabilities (Kozma et al., 2009). It also gives people with developmental disabilities more autonomy (Kilroy et al., 2015; King et al., 2017). However, community living does not automatically bring all these benefits unless the support system is organized to facilitate community engagement of adults with developmental disabilities. Many researchers suggested that personal support networks are important for people with developmental disabilities when it is a matter of community living. Personal support networks (sometimes also termed circles of support or support networks) are a strategy to formalize support embedded in informal networks, meaning a group of people come together voluntarily, with varying levels of formality, to support an individual through relationships of trust and intimacy (Macadam & Savitch, 2015). An individual with a disability identifies the people they want to be in their personal support network; thus, it gives an individual with a disability more control over all areas of their life. However, the role of natural and formal support is pivotal within the personal support network. Future studies can explore the feasibility and effectiveness of this form of support in Canada.

A key finding of this study is that many formal support providers in Canada play a critical role in establishing the social network of individuals with developmental disabilities. Formal support providers are using two approaches to foster natural connections: social inclusion and social capital. In the social inclusion approach, service providers establish the link between social networks and individuals with developmental disabilities, whereas, in the social capital approach, formal support providers assist people with developmental disabilities in building their capacity to form relationships and support networks. Evidence suggests that the social capital approach is more likely to be effective in promoting independent living. However, it involves lots of time and resource commitments for service providers to practice (Clement et al., 2023; McConkey & Collins, 2010), reinforcing the need for government to allocate more resources in this area.

### Implications for Policy, Practice and Research

The findings of this study have policy, practice and research implications in disability fields. Service providers affirmed the unique role of natural support providers for adults with disabilities in Canada. Service providers also outlined their roles and limitations to provide optimal care to adults with developmental disabilities, including often significant systemic and personal boundaries that limit the scope or contributions of formal supports. Although boundaries are often put in place for the safety of support provider and recipient, strict policies (and to-the-letter application of such policies) can dehumanize the personal connections inherent in support provision and receipt (Busch et al., 2019), and can impede people with developmental disabilities from receiving the type of the support they truly need to live their desired life. We would encourage both policymakers and support providing organizations to strive to uphold individual needs and choices and promote relationship development among provider and recipient, while still ensuring safety for all involved. This may mean revisiting policies and procedures, or their application to truly assess if boundaries are, indeed, necessary or if flexibility in formal support provision may be accommodated to meet each individual’s support needs. Similarly, we encourage policymakers to accommodate a level of flexibility in their provision of support funding. If, for example, supports desired by the individual are beyond the scope, expertise, or capacity of those in the formal support system, but could be offered by one’s natural support network, policymakers should enable opportunities for supports funding to bolster and empower natural support options. This flexibility in financing for supports based on individual desire is an innovative model that is already being applied in some places in Canada (Stainton et al., 2013) and abroad (DeCarlo et al., 2019; Fleming et al., 2016; Reddihough et al., 2016). Further research might explore the impact on individual and family quality of life when funding for supports are shifted from a systemic, one-size-fits-all model to enable more individualized and self-directed supports funding.

Beyond this, the present study participants affirmed the unique role that natural supports play in one’s life, and thus, in addition to enabling flexibility of formal support funds (to go to natural support networks if desired), further investments could be made to directly support the creation and maintenance of natural support networks for persons with disabilities. This funding may also include support for formal support providers such as “facilitators” or “connectors” who can enable persons with developmental disabilities and their families identify opportunities in the community. Further research could explore with persons with developmental disabilities and their families how they might enable and enhance the natural supports in their lives.

### Limitations

This study has a number of limitations. First, most of our participants were recruited based on the recommendations of the advisory group/team members of this study, which may have created selection bias towards a specific group of formal support providers. Second, a majority of the participants were from two provinces (British Columbia and Ontario). This was because the author team, based in Ontario and British Columbia, used our local networks to support recruitment. Interviewing more formal support providers from other provinces could offer different and important insights. Third, most of the participants held senior positions at the time of the interview, although they had all worked as front-line service providers for a long time before being promoted to leadership roles. Because many participants were not actively providing direct support at the time of the interview, they may have offered experiences that do not completely align with the current implementation of direct supports, or reflections that are more high-level or abstract than those that might come from someone still involved in day-to-day support provision. Finally, it is important to note that the results represented only the views of formal support providers, which may not fully align (or could potentially contradict) the views of natural support providers. Future research on this topic must include the voices of adults with developmental disabilities and their families to get a deeper understanding of this phenomenon – our efforts to capture these perspectives on this topic are currently underway. Despite these limitations, the findings of this study contribute to the growing literature that attempts to find a balance between formal and natural support and shed light on how to integrate these two supports for adults with developmental disabilities and their families.

## Conclusion

The results of this study highlight the importance of both formal and natural supports for adults with developmental disabilities and their families in Canada. Co-existence and seamless interactions between these two support systems are critical to increasing community participation of individuals with developmental disabilities. These findings have important implications for how formal supports need to be organized so that natural supports of persons with developmental disabilities feel supported. The government needs to allocate resources to incentivize and enable both types of support. Direct funding to individuals with developmental disabilities themselves is critical to enable them to access the right type of support for them at the right time. Practitioners should help people with developmental disabilities with knowledge and skills on how to build and maintain connections with people in their society. Future studies must incorporate the perspectives of adults with developmental disabilities and their families to better understand their support needs.

## Footnotes

1 A Canadian federal government plan that enables individuals and families to save for the future, tax-free.
